# Optimization of genetic distance threshold for inferring the CRF01_AE molecular network based on next-generation sequencing

**DOI:** 10.3389/fcimb.2024.1388059

**Published:** 2024-05-22

**Authors:** Lijuan Hu, Bin Zhao, Mingchen Liu, Yang Gao, Haibo Ding, Qinghai Hu, Minghui An, Hong Shang, Xiaoxu Han

**Affiliations:** ^1^ State Key Laboratory for Diagnosis and Treatment of Infectious Diseases, National Clinical Research Center for Laboratory Medicine, The First Hospital of China Medical University, China Medical University, Shenyang, China; ^2^ National Health Commission (NHC) Key Laboratory of AIDS Prevention and Treatment, National Clinical Research Center for Laboratory Medicine, The First Hospital of China Medical University, China Medical University, Shenyang, China; ^3^ Laboratory Medicine Innovation Unit, Chinese Academy of Medical Sciences, Shenyang, China; ^4^ Key Laboratory of AIDS Immunology of Liaoning Province, Shenyang, China; ^5^ Collaborative Innovation Center for Diagnosis and Treatment of Infectious Diseases, Hangzhou, China

**Keywords:** HIV, genetic distance, next-generation sequencing, molecular network, CRF01_AE

## Abstract

**Introduction:**

HIV molecular network based on genetic distance (GD) has been extensively utilized. However, the GD threshold for the non-B subtype differs from that of subtype B. This study aimed to optimize the GD threshold for inferring the CRF01_AE molecular network.

**Methods:**

Next-generation sequencing data of partial CRF01_AE *pol* sequences were obtained for 59 samples from 12 transmission pairs enrolled from a high-risk cohort during 2009 and 2014. The paired GD was calculated using the Tamura-Nei 93 model to infer a GD threshold range for HIV molecular networks.

**Results:**

2,019 CRF01_AE pol sequences and information on recent HIV infection (RHI) from newly diagnosed individuals in Shenyang from 2016 to 2019 were collected to construct molecular networks to assess the ability of the inferred GD thresholds to predict recent transmission events. When HIV transmission occurs within a span of 1-4 years, the mean paired GD between the sequences of the donor and recipient within the same transmission pair were as follow: 0.008, 0.011, 0.013, and 0.023 substitutions/site. Using these four GD thresholds, it was found that 98.9%, 96.0%, 88.2%, and 40.4% of all randomly paired GD values from 12 transmission pairs were correctly identified as originating from the same transmission pairs. In the real world, as the GD threshold increased from 0.001 to 0.02 substitutions/site, the proportion of RHI within the molecular network gradually increased from 16.6% to 92.3%. Meanwhile, the proportion of links with RHI gradually decreased from 87.0% to 48.2%. The two curves intersected at a GD of 0.008 substitutions/site.

**Discussion:**

A suitable range of GD thresholds, 0.008-0.013 substitutions/site, was identified to infer the CRF01_AE molecular transmission network and identify HIV transmission events that occurred within the past three years. This finding provides valuable data for selecting an appropriate GD thresholds in constructing molecular networks for non-B subtypes.

## Introduction

The HIV molecular network based on the similarity of the viral gene serves various purposes, such as reconstructing HIV transmission history (Hue et al., 2004), real-time monitoring of network expansion, and assessing individual HIV transmission risk for precise intervention and control (Han et al., 2020). The HIV-1 pol gene, widely used for routine HIV drug resistance analysis, has now become a common resource for constructing HIV molecular networks due to its accessibility and abundant data resources (Hue et al., 2004). The crucial parameter in constructing molecular networks is the cutoff value of genetic distance (GD), which reflects the similarity between individuals potentially connected and determines the number of transmission clusters and the number of sequences within each cluster (Junqueira et al., 2019). Pairwise GD calculated using the Tamura-Nei 93(TN93) model provides a fast computation that takes into account variations in base composition and transition/transversion rate (Hightower et al., 2013). It has been recommended by the Centers for Disease Control and Prevention in the United States (National Center for HIV/AIDS, Viral Hepatitis, STD, and TB Prevention and Division of HIV/AIDS Prevention, 2018) and China (Prevention & center, 2019) for inferring the HIV molecular network.

CRF01_AE is the first confirmed circulating recombinant form (CRF) of HIV-1 and is the second most prevalent CRF worldwide (Hemelaar et al., 2020). It is endemic in Europe, North America, central and west Africa, Asia, and Australia, accounting for approximately 5% of global HIV-1 strains (Hemelaar et al., 2011). In Southeast Asia, CRF01_AE is the predominant strain, accounting for around 79% (Hemelaar et al., 2011). In China, CRF01_AE is the second most prevalent strain and has become the dominant strain among men who have sex with men (MSM) (Li, 2016). Recent studies have focused on investigating the epidemic pattern and characteristics of CRF01_AE by constructing CRF01_AE molecular network (Chang et al., 2018; Zhang et al., 2020; Zhao et al., 2021b).

The construction of an HIV molecular network is based on an understanding of virus evolution. However, the presence of numerous HIV-1 subtypes and CRFs worldwide poses a challenge. There are significant variations in HIV epidemic strains, transmission dynamics, surveillance practices, and treatment levels across different countries and regions. Additionally, different HIV-1 subtypes may exhibit varying rates of evolution (Raghwani et al., 2018). For instance, It has been observed that the evolution of the *pol* region in subtype B does not exceed 0.01 substitutions/site (subs/site) (i.e., there are 10 base differences in 1000 nucleotides) over 10 years in a natural setting (Hightower et al., 2013). It has also been found that a GD threshold range of 0.01-0.02 subs/site, with an optimal threshold of 0.0175, could be employed to construct an HIV-1 subtype B molecular network (Wertheim et al., 2017). However, our team found that the optimal GD threshold for constructing the CRF01_AE molecular network was 0.007 subs/site, which differs from that for subtype B (Zhao et al., 2021a). Thus, using the GD threshold for subtype B to infer the CRF01_AE molecular network may introduce biases in the clustering rate and other network parameters.

Therefore, it is crucial to investigate the evolution of the CRF01_AE *pol* gene to determine the appropriate GD threshold range for inferring the CRF01_AE molecular network. In this study, we selected MSM transmission pairs infected with CRF01_AE from a high-risk cohort and conducted subsequent follow-up observations. We employed next-generation sequencing (NGS) technology to acquire partial HIV pol sequences and calculated the GD between them. These GD values were then compared to infer a reasonable GD threshold range for constructing the CRF01_AE molecular network, which is a valuable tool in predicting potential recent HIV transmission events.

## Materials and methods

### Study population

#### Longitudinal samples of HIV transmission pairs in the acute phase

From 2009 to 2014, a high-risk cohort of HIV-negative MSM was established through voluntary testing and counseling clinics in Shenyang. HIV transmission pairs were identified through regular follow-up, each of which included at least one case of acute HIV infection (AHI) (Zhang et al., 2021). The inclusion criteria for research subjects were as follows: 1) age ≥18 years, 2) confirmed HIV infection with only HIV-1 CRF01_AE strain, 3) self-reported HIV transmission relationship supported by a phylogenetic branch with a high bootstrap value (details provided in phylogenetic analysis). 4) HIV treatment-naïve before HIV diagnosis and during the follow-up period, and 5) collection of at least one plasma sample from all participants at baseline. This data from HIV transmission pairs was used to establish the appropriate GD threshold range for inferring the CRF01_AE molecular network.

An individual was considered to have AHI when the following conditions need to be met (Zhang et al., 2021): (1) the interval between the last HIV antibody-negative and first confirmed HIV-1 antibody-positive was less than 6 months, (2) the HIV antibody test was negative but the HIV RNA test was positive, or (3) the optical density of the initial HIV antibody test is between ranged 0.5 and 1.0, and then increased during follow-up. During the follow-up period, all individuals were ultimately confirmed to be infected with HIV-1. The estimation of the infection time for AHI was based on the Fiebig stage (Fiebig et al., 2003). The estimated time of infection for individuals in Fiebig stage VI and those with longstanding HIV infection was calculated as the average of the interval between the last negative HIV antibody test and the first confirmed positive HIV antibody test.

#### HIV-1 positive samples from a cross-sectional survey

The HIV-1 CRF01_AE *pol* sequence (HXB2:2,253-3,300) and the results of the HIV-1 Limiting Antigen Avidity assays (Recent HIV infection [RHI] discrimination experiment) were collected from all individuals newly diagnosed with HIV in Shenyang from 2016 and 2019 (Zhao et al., 2021b). The data from this cohort was utilized to evaluate the efficacy of the CRF01_AE molecular network in inferring recent transmitted events in the real world, employing the established GD threshold range. The study was approved by the Institutional Review Board of China Medical University.

### Sanger sequencing and phylogenetic analysis to verify the transmission pair

The HIV *pol* sequence (HXB2:2,253-3,300) was obtained using an in-house method (Zhao et al., 2021a). The Subtyping of the sequences was determined by performing Neighbor-joining (NJ) tree analysis which involved comparing the sequences with reference sequences from the Los Alamos database [“ LANL. HIV Sequence Database. In. http://www.hiv.lanl.gov/ (Date of access:02/08/2019).”]. Detailed sequence analysis procedures have been described previously (Zhao et al., 2021a). To verify the transmission relationship between transmission pairs, an NJ tree was constructed. If the donor and recipient sequences of a “transmission pair” formed a branch with a bootstrap value greater than 0.95, and no other sequences were inserted within the branch, it was preliminarily classified as a transmission pair (Verhofstede et al., 2019). The direction of transmission within a confirmed transmission pair was determined based on epidemiological investigation.

### NGS for inferring CRF01_AE GD threshold range

HIV *pol* region (HXB2:2868-3320) was reverse transcribed into cDNA using Super Script^®^ III First-Strand Synthesis Super Mix Kit (Invitrogen, Carlsbad, CA, USA). The fragment was then amplified using KOD high fidelity enzyme (KOD, Toyobo, Japan), purified with Agencourt AM Pure XP Kit (Beckman-Coulter, Brea, CA, USA) according to the provided instructions, and quantified using a QUBIT fluorescence quantification analyzer (ThermoFisher Scientific, USA). The library was constructed using Truseq Nano DNA HT Library Prep protocol (Illumina, San Diego, CA, USA), which included end modification and fragmentation, 3’ “A” tail modification, connection adapter, library amplification, library qPCR quantification, library standardization, using Miseq Reagent V3-600cycles kit (Illumina, San Diego, CA, USA) for Miseq indexing.

To analyze the data packets generated by NGS, the Oracle VM Virtual Box-5.2.22 software package was used to create a Virtual environment under the Windows operating system. QIIME2 Core-2018.4 (1525276946) was then employed to perform the analysis. The sequence and quantity of HIV-1 quasispecies were reported for each sample. The Fast QC (version 0.11.7) was used to analyze the per-base sequence quality and per-sequence quality scores at each sampling point (Ji et al., 2018). Quasispecies with a proportion of ≥1% in each sample were selected for subsequent analysis. The sequences were aligned to the HXB2 reference and then trimmed to a length of 453 base pairs.

### Computing pairwise genetic distance and molecular network analyses

The GD between any two sequences was calculated using the TN93 model and Gamma Distributed model of Mega7.0.14 (Kumar et al., 2016). Molecular networks based on the nucleotide GD were constructed using HIV-TRACE (Kosakovsky Pond et al., 2018). A linkage was established whenever the pairwise GD fell below the GD threshold. During pairwise GD calculation, all nucleotide ambiguities were resolved, and only sequences with less than 5% ambiguities were included in the analysis (Wertheim et al., 2017).

### Statistical analysis

Analysis of Variance (ANOVA) was applied to compare the GDs between groups. GraphPad Prism 8 (GraphPad, La Jolla, CA, United States) was used for all statistical analyses and data visualization. P<0.05 was considered statistically significant.

## Results

### Study population

**Figure 1:**
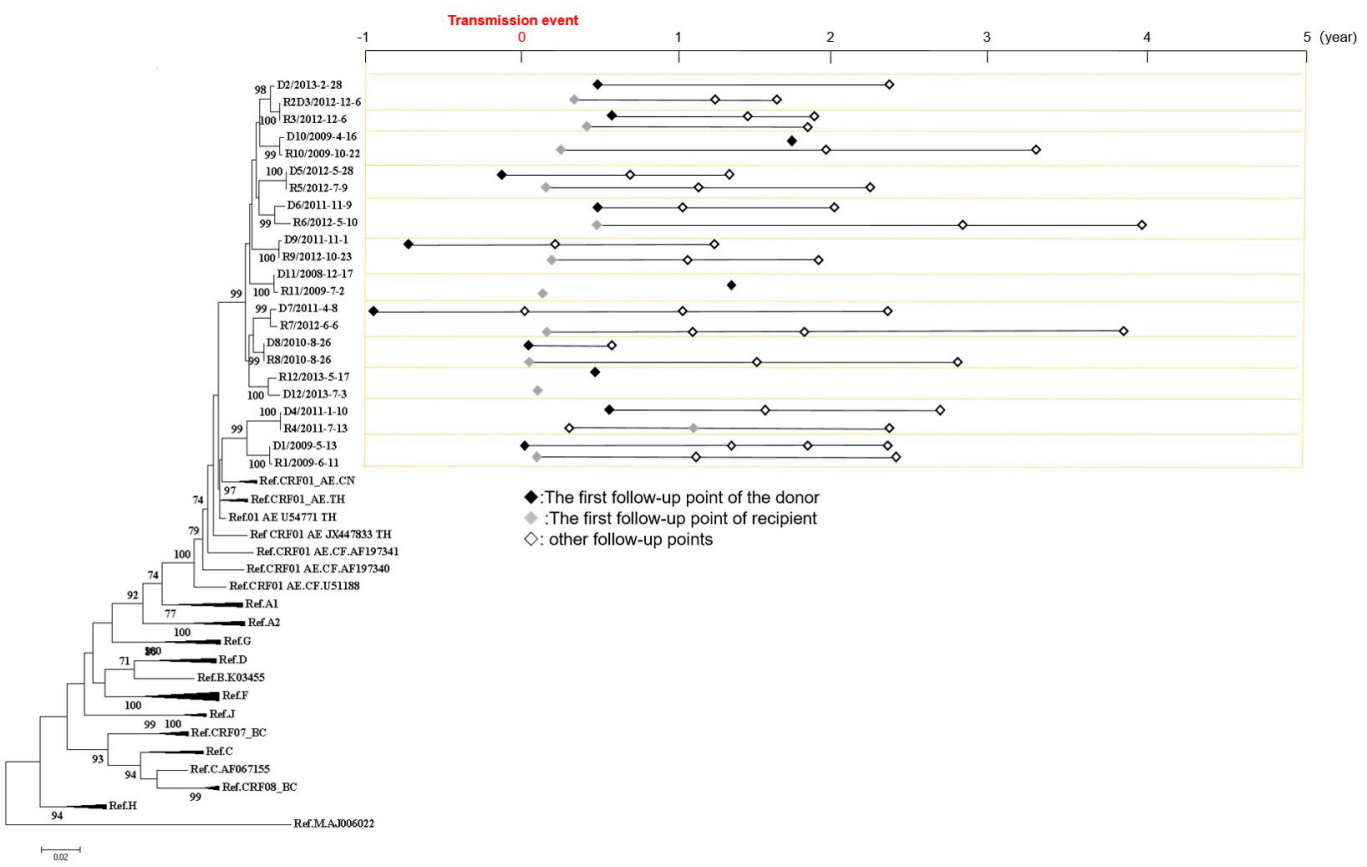
Phylogenetic analyses of pol gene and the follow-up sampling points from 12 HIV transmission pairs involving men who have sex with men. On the left side, a Neighbor-joining tree was constructed based on Sanger sequences of HIV pol gene (HXB2:2253-3311). On the right side, the follow-up sampling points were depicted. The letter “D” denotes the donor, the letter “R” denotes the recipient, and the numbers indicate the serial number of transmission pairs. R2+R2D3+D3 were two transmission pairs consisting of three individuals, where R2D3 was the same individual who served as both the recipient of D2 and the donor of R3.

In this study, a total of 12 transmission pairs among MSM were included, resulting in a sample size of 23 individuals (R2+R2D3+D3 were two transmission pairs consisting of three individuals, where R2D3 was the same individual who served as both the recipient of D2 and the donor of R3, **Figure 1**). The phylogenetic analysis revealed a median bootstrap value of 100 (range: 98-100) for all transmitted pairs. The median age of all the 23 individuals was 33 years (range: 19-46 years). A total of 59 samples were collected from these 23 individuals, and the follow-up period covered 606 person-months. Among the individuals, 17 were identified to have AHI (D1, R2D3, D5, D7-9, D12, R1, and R4-12), which corresponds to the period between 1 and 176 days after HIV infection, with a mean time of 63 days (**Figure 1**).

**Figure 1** illustrates the distribution of sampling points relative to the transmission events. Within the dataset, 6.8% (4/59) of the sampling points were collected from the donors of the transmission pairs within one year before the transmission event, while 37.3% (22/59) were collected within one year after the transmission event. Furthermore, 35.6% (21/59) of the sampling points were within 1-2 years after the transmission events, 15.3% (9/59) were within 2-3 years after the transmission events, and 5.1% (3/59) were within 3-4 years after the transmission events.

### Evaluation of NGS data quality

The average base quality value (Q30) indicates that there is one incorrect base recognition per 1000 bases, indicating a correct recognition rate of 99.9%call per 1000 bases, resulting in a 99.9% accuracy rate for base recognition. Q30 of the original NGS data for each sample was 82.7% in this study (ranging from 70.1% to 92.7%). Following sequence alignment and screening, the average per-base sequence quality value was 83% (range: 73% to 92%). Additionally, the average per sequence quality score was 84% (range: 78% to 89%). Each sample exhibited an average of 4 quasispecies (range: 1 to 23). Moreover, the average number of reads per sequence was 49,969 (range: 118 to 411,775) (**Supplementary Figure 1**).

### Correlation between GD threshold and the duration of HIV infection

**Figure 2:**
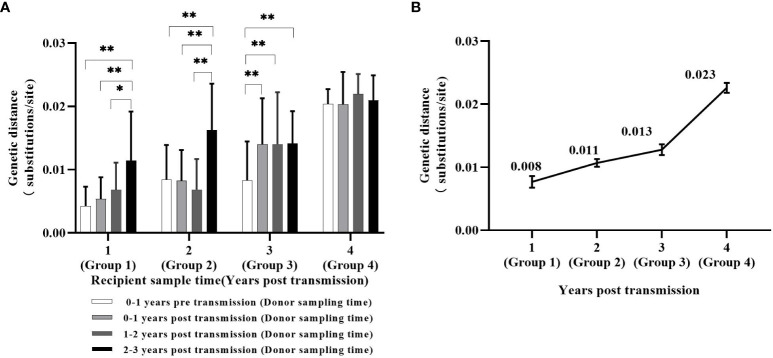
The average paired genetic distances (GD) between recipient and donor sequences within the same transmission pair. **(A)** Sixteen sets of the average paired GD between pol sequences (HXB2:2868-3320) of the recipient and the donor within the same transmission pair based on the time of HIV infection of the recipient. **(B)** The average paired GD between pol sequences of the recipient and donor within the same transmission pair during the 1-4 years following the occurrence of HIV transmission events. *P< 0.05, **P < 0.01.

The average GD between sequences of the donor and recipient from the same transmission pairs was calculated. Sixteen sets of GD values were obtained and divided into four groups based on the time of HIV infection of the recipient within the transmission pairs: 1-4 years post-transmission (YPT) (Group1:n=9 transmission pairs, 29 samping points; Group2:n=9 transmission pairs, 39 samping points, Group3:n=7 transmission pairs, 25 samping points Group4:n=4 transmission pairs, 11 samping points) (**Figure 2A**). Each group consisted of four values representing the GD between the recipient’s sequence within a specific period (1-4 YPT) and the sequences of the donor at four different follow-up points (1 year before transmission [YBT], 1 YPT, 1-2 YPT and 2-3 YPT) (**Figure 2A**).

In Groups 1 and 2, The average GD between the recipient sequences and the donor sequences at 2-3 YPT was significantly higher than the GD values between the recipient sequences and the donor sequences at other follow-up points (Group 1: 2-3 YPT: 0.011 subs/site vs. 1 YBT: 0.004 subs/site, 1 YPT: 0.005 subs/site and 1-2 YPT: 0.007 subs/site, P<0.05; Group 2: 2-3 YPT: 0.016 subs/site vs. 1 YBT: 0.008 subs/site, 1 YPT: 0.008 subs/site and 1-2 YPT: 0.007 subs/site, P<0.05). However, In Group3, the average GD between the recipient sequence and the donor sequences at 1 YBT was significantly lower than the GD values between the recipient sequences and the donor sequences at other follow-up points (1 YBT:0.008 subs/site vs. 1 YPT/1-2 YPT/2-3 YPT: 0.014 subs/site, P<0.05). There was no significant difference among the four GD values in Group 4 (1 YBT/1 YPT: 0.020subs/site, 1-2 YPT: 0.022subs/site, and 2-3 YPT: 0.021 subs/site) (**Figure 2A**).

In summary, when the transmission event occurs within 1 year (Group1), 1-2 years (Group2), 2-3 years (Group3), and 3-4 years (Group4), the average paired GD between the recipient sequences and the donor sequences within the same transmission pair were 0.008 (95%CI:0.007-0.009), 0.011(95%CI:0.010-0.011), 0.013(95%CI:0.012-0.014), and 0.023(95%CI:0.022-0.023) subs/site, respectively (**Figure 2B**). The differences in GD values between any two groups were statistically significant (P<0.01).

### The effectiveness of GD thresholds in identifying transmission pairs

**Figure 3:**
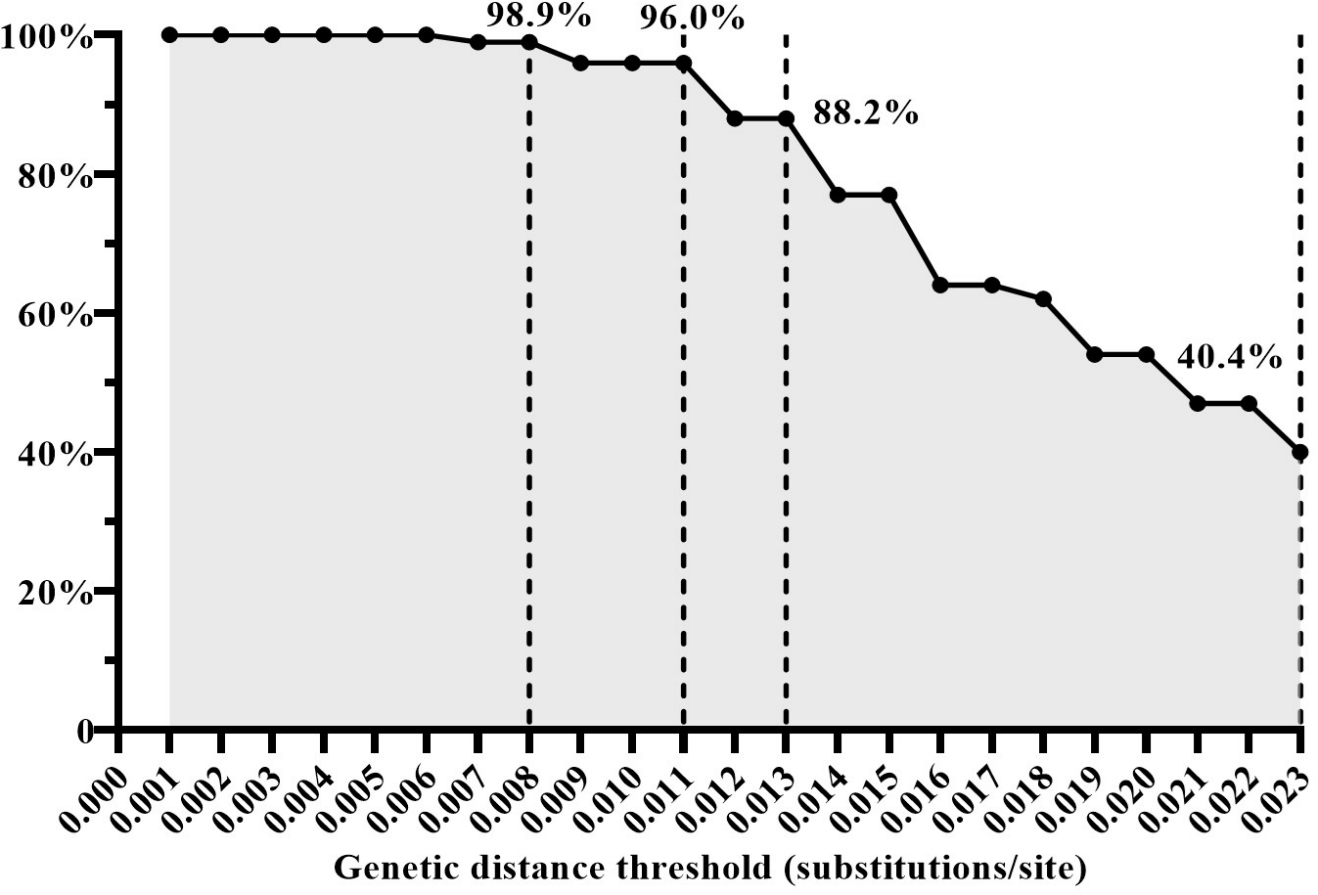
The proportion of genetic distance (GD) between the sequences of the recipient and donor from the same transmission pairs among the GD between all randomly paired sequences under the condition of different GD thresholds.

To assess the effectiveness of the four inferred GD thresholds (0.008, 0.011, 0.013, and 0.023 subs/site) in identifying HIV transmission events (i.e. discerning transmission pairs), we analyzed the proportion of the GD value between the sequences of the recipient and donor within the same transmission pairs among the GD between all randomly paired sequences. A total of 224 quasispecies from 12 transmission pairs were filtered and analyzed in this study. For instance, when employing a GD threshold below 0.008 subs/site, 98.9% (438/443) of the GD values were from the same transmission pairs. Similarly, with a GD threshold below 0.011 subs/site, 96.0% (549/572) of the GD values were identified as originating from the same transmission pairs. When the GD threshold was set below 0.013 subs/site, 88.2% (637/722) of the GD values were associated with the same transmission pairs. However, using a GD threshold below 0.023 subs/site, only 40.4% (1021/2530) of the GD values were linked to the same transmission pairs (**Figure 3**). Based on these findings, a GD threshold of 0.013 subs/site could be considered as the upper limit for the optimal GD threshold in inferring CRF01_AE molecular network. This threshold demonstrated a high proportion of GD values originating from the same transmission pairs, thus providing a reliable indication of identifying HIV transmission events.

### Evaluation of the impact of different GD thresholds in practical applications

Finally, we assessed the effectiveness of the three estimated GD thresholds (0.008, 0.011, and 0.013 subs/site) in identifying new transmission events (i.e. RHI) in real-world scenarios. According to the previous study (Zhao et al., 2021b), a total of 2019 CRF01_AE sequences from newly diagnosed HIV-1 infections were collected in Shenyang from 2016 to 2019 (2016:n=471; 2017:n=554; 2018:n=517; 2019:n=477). 680 (33.7%) were identified as RHI (2016: n=152; 2017: n=185; 2018: n=200; 2019: n=143).

**Figure 4:**
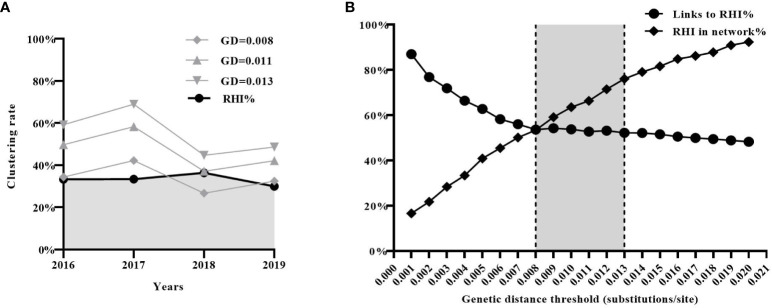
Evaluation of the effectiveness of the inferred three genetic distance (GD) thresholds (0.008, 0.011, and 0.013 subs/site) in real-world scenarios. **(A)** The effectiveness of the three inferred GD thresholds in identifying recent HIV infection. **(B)** The analysis of sensitivity (the proportion of RHI in the network) and specificity (the proportion of links connecting to RHI) of GD threshold. RHI, recent HIV infection.

Molecular transmission networks were constructed using the three estimated GD thresholds. Individuals included in the network constructed with a GD threshold of 0.008 subs/site were considered to acquire newly HIV infection within a year. The proportion of individuals in this network (2016:34%; 2017:42%; 2018:27%; 2019:32%) closely matched the proportion of RHI determined by the HIV-1 Limiting Antigen Avidity assays (2016:33%;2017:33%;2018:36%;2019:30%)(**Figure 4A**). Furthermore, it was observed that the proportion of RHI in the network gradually increased (RHI in the network/all RHI, from 16.6% to 92.3%) with an increment in the GD threshold (from 0.001 to 0.02 subs/site). However, the proportion of links connecting to RHI in the network gradually decreased (from 87.0% to 48.2%), indicating the introduction of more false links into the network. The two curves intersected at a GD threshold of 0.008 subs/site, suggesting that 0.008 subs/site could be considered as the optimal GD threshold in the CRF01_AE molecular network (**Figure 4B**).

Based on the above results, the optimal GD threshold range for inferring the CRF01_AE molecular network could be 0.008 to 0.013 subs/site. This range provides a balance between accurately identifying RHI and minimizing the inclusion of false links within the network.

## Discussion

This study analyzed NGS data of the pol gene from 12 MSM transmission pairs at different infection times to determine the GD threshold range for inferring the molecular network of CRF01_AE, enabling the inference of recent HIV transmission events occurring within different infection time ranges (1-3 years). This study offers crucial reference data for selecting an appropriate GD threshold to construct the CRF01_AE molecular transmission network in real-world scenarios.

Our previous study also found that the optimal GD threshold (0.007subs/site) used to identify recent transmission events in constructing the CRF01_AE molecular network differed from the subtype B (0.015subs/site) (Zhao et al., 2021a). In this study, we found that when HIV CRF01_AE subtype transmission event occurred within one year, the average paired GD between recipients and donors sequences of CRF01_AE within the same transmission pair was determined to be 0.008 (95%CI:0.007-0.009) subs/site. This finding further supported the rationale for the optimal GD threshold (0.007subs/site) for the CRF01_AE molecular network based on the previous study (Zhao et al., 2021a), indicating that the molecular transmission network built using this threshold can accurately predict recent HIV transmission events occurring within one year. There were significant differences in the evolutionary rates of different HIV-1 subtypes (Patino-Galindo and Gonzalez-Candelas, 2017). As the prevalence increases, the evolutionary rate of HIV-1 slows down (Maljkovic Berry et al., 2007). This phenomenon can be attributed to transmissions occurring during the early stages of host infection when the selective pressures of the immune system have minimal impact, leading to a decrease in the rate of HIV-1 evolution at the population level. Therefore, this study also suggests that the low GD threshold observed for CRF01_AE may be due to its rapid spread among MSM in China (An et al., 2021).

Our results also revealed that GD between sequences increases with the extension of infection interval, indicating virus evolution over time. While larger GD can identify a wider range of transmission event periods, it also increases the risk of including individuals without transmission relationships. When the GD thresholds were set below 0.008 substitutions/site, 98.9% (438/443) of the GD values originated from the same transmission pairs. This finding strongly suggests that when the GD between two pol sequences is ≤0.008 substitutions/site, it is highly likely that they have a transmission relationship. Conversely, as the GD threshold increased, the proportion of GD values between recipient and donor sequences from the same transmission pair gradually decreased. Specifically, when the GD thresholds were set below 0.023 subs/site, only 40.4% of GD values originated from the same transmission pairs. These results indicated that it is more reliable to infer the CRF01_AE molecular network using a GD threshold range of 0.008-0.013 sub/site to determine HIV transmission events that occurred within the past three years.

The practical application of the inferred GD threshold range for the CRF01_AE subtype was also validated in the real world. Firstly, we found that only the proportion of individuals in the molecular network using a GD threshold of 0.008 substitutions/site closely matched the proportion of RHI determined by the results of HIV-1 Limiting Antigen Avidity assays. This result is easily understandable, as the HIV-1 Limiting Antigen Avidity assays theoretically identify RHIs who have been infected with HIV for approximately six months, while the CRF01_AE molecular network using a GD threshold of 0.008 substitutions/site identifies individuals who experienced transmission events within one year. Secondly, our findings revealed that as the GD threshold increased, the proportion of RHIs included in the network (sensitivity) gradually increased, while the proportion of links connecting to RHIs (specificity) in the network gradually decreased. However, when the GD threshold was set to 0.008 substitutions/site, the molecular network achieved the optimal balance between sensitivity and specificity for identifying RHIs.

The current Guidelines in China recommended a threshold range of 0.005 substitutions/site and 0.015 substitutions/site for inferring molecular networks of all subtypes (Prevention & center, 2019). These thresholds are used to speculate on HIV transmission events occurring within 2-3 years and 5-15 years. Our research findings are not contradictory to the guidelines; instead, they provide a more accurate GD threshold range for constructing the CRF01_AE molecular network and a time frame for the occurrence of HIV transmission events. For instance, our results suggested that using a GD threshold of 0.005 substitutions/site may infer the molecular transmission network of CRF01_AE for HIV transmission events occurring within one year. However, this threshold may underestimate the scale of the molecular transmission network, resulting in fewer clusters. On the other hand, using a GD threshold of 0.015 substitutions/site may infer a molecular transmission network for CRF01_AE that overestimates the scale of the network, including more unrelated individuals.

Lastly, NGS technology can overcome the limitations of Sanger sequencing, providing improved sensitivity and compensating for its inherent randomness. In the presence of immune pressure, HIV produces multiple mutates and quasispecies (Poon et al., 2007). However, the Sanger sequencing method can only obtain a single consensus sequence that represents the dominant HIV quasispecies in the sample (Lapointe et al., 2015), which is subject to randomness and uncertainty. NGS, on the other hand, offers deeper coverage, typically generating thousands to tens of thousands of reads per sample (Simen et al., 2009; Ram et al., 2015). This increased coverage allows for the detection of low-frequency variations (Barzon et al., 2011). Therefore, GD values calculated based on sequences obtained from NGS are highly likely to be greater than those calculated based on sequences obtained from Sanger sequencing, aiming to be closer to the true GD value. Notably, we observed the presence of more than 10 quasispecies (specifically, 14 and 23 quasispecies) at two sampling points in this study. This result suggests that the evolution of HIV under immune pressure due to a prolonged duration of infection may have contributed to the observed quasispecies diversity.

This study still has certain limitations. Firstly, the evolution rate of the HIV pol sequence could be underestimated. due to the analysis focusing on only half the length of the pol sequence (HXB2: 2868-3320) typically used for HIV drug resistance analysis. Secondly, This study focuses on the MSM in a moderately prevalent HIV city in Northeast China. Whether it can represent the transmission patterns of HIV-infected individuals in other regions or the high-risk populations who acquire HIV through other routes (such as the heterosexual transmission or intravenous drug user) requires further research for confirmation. Lastly, the limited number of acute HIV transmission pairs, roughly estimated time of HIV infection, and some of lower-quality NGS sequences were potential sources of bias and confounding factors that cannot be avoided. These factors may lead to overestimation or underestimation of the final results.

## Conclusion

Based on the analysis of NGS data from HIV transmission pairs, this study has established a valid GD threshold range (0.008-0.013 subs/site) for inferring the CRF01_AE molecular network, enabling the accurate identification of HIV transmission events that happened within the past three years. This study provided robust data support for selecting the appropriate GD threshold when constructing molecular networks for non-B subtypes.

## Data availability statement

The datasets presented in this study can be found in online repositories (GenBank accession numbers PP696627 to PP696852 and PP696864 to PP696886).

## Ethics statement

The studies involving humans were approved by The Institutional Review Board of China Medical University. The studies were conducted in accordance with the local legislation and institutional requirements. The participants provided their written informed consent to participate in this study.

## Author contributions

LH: Formal analysis, Writing – original draft, Investigation. BZ: Supervision, Writing – original draft, Investigation, Visualization, Writing – review & editing. ML: Methodology, Writing – review & editing. YG: Methodology, Software, Writing – review & editing. HD: Data curation, Writing – review & editing. QH: Data curation, Writing – review & editing. MA: Methodology, Software, Writing – review & editing. HS: Conceptualization, Project administration, Resources, Writing – review & editing. XH: Conceptualization, Funding acquisition, Supervision, Writing – review & editing.

## Supplementary material

Supplementary Figure 1 Quality Evaluation of next-generation sequencing (NGS). **(A)** The per base sequence quality and per sequence quality scores of NGS data. **(B)** The number of quasispecies and reads of each sample.
