# Supplementary figures and images for: Optimization of genetic distance threshold for inferring the CRF01_AE molecular network based on next-generation sequencing
Source: Front Cell Infect Microbiol. 2024 May 22;14:1388059. doi: 10.3389/fcimb.2024.1388059 (PMC11155296; doi:10.3389/fcimb.2024.1388059)

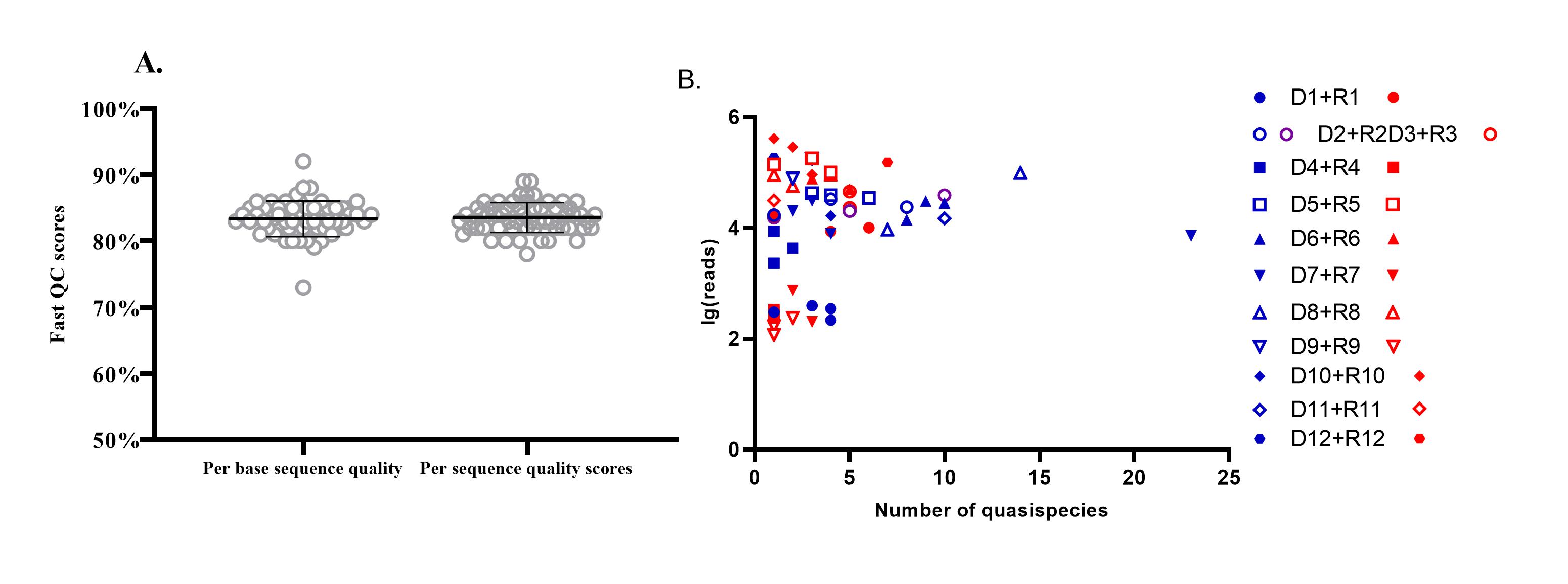

Supplement: Supplementary Figure 1 — Quality Evaluation of next-generation sequencing (NGS). (A) The per base sequence quality and per sequence quality scores of NGS data. (B) The number of quasispecies and reads of each sample. [file Image_1.jpeg]
